# GENESIS 3.0 functionality enhanced by interface to multiple types of database

**DOI:** 10.1186/1471-2202-12-S1-P15

**Published:** 2011-07-18

**Authors:** Hugo Cornelis, Allan D Coop, Armando L Rodriguez, Dave Beeman, James M Bower

**Affiliations:** 1 Department of Neurophysiology, Catholic University of Leuven, Leuven, Belgium; 2 Merindah Energy Ltd, Freemansreach, NSW, 2756, Australia; 3 Research Imaging Institute, UT Health Science Center at San Antonio, San Antonio, TX, USA; 4 Department of Electrical, Computer, and Energy Engineering, University of Colorado, Boulder, CO 80309, USA

## 

An important feature of the GENESIS 3 simulator (G-3) is that its use is structured following an ideal user-workflow with a focus on actual experimental paradigms rather than the process of computational simulation by itself. This organization reflects the likely growing importance of linking models to real experiments and experimental data. As a consequence, the software architecture of the G-3 simulator allows it to be connected directly to independent databases that store models, experimental protocols, libraries of numerical solvers, repositories of results and aggregated analysis, and ultimately, tutorials and publications, as well as real time experimental protocols such as dynamic clamping procedures. In this paper, we present and explain the details of the database interfaces that support this functionality and show examples of data-driven modeling methods.

The ideal user-workflow consists of 5 steps: (1) Construct model, (2) Design experiment, (3) Run simulation, (4) Check and analyze results, including experimentally, and (5) Iterate. In principle, each of these steps provides the foundation for databases that collectively define the scope and content of a modeling project.

We provide three concrete and already implemented examples of the relationship between steps in the ideal user workflow and the databases that support them.

*1. Construct Model:* The G-3 software component that implements this first step in the ideal user workflow is called the Model-Container. It natively supports a dedicated data format (NDF) for computational models in neuroscience. The Model-Container has a built-in database of NDF models and also interfaces with arbitrary external model representations, including currently both NeuroML and NineML protocols. We show how to add new computational models to this database as well as how to interface to new model databases.

*2. Design Experiment:* The second step in the ideal user workflow connects the G-3 simulator to a database of generic experimental protocols such as current injection and current or voltage clamp. These protocols can be adjusted by a user to define specific experimental procedures. Note that these procedures are stored independently of the specification of the computational model, and can also, in principle, be obtained from any experimental source of data.

We will demonstrate how the separation of the steps for model construction and experimental design allow, in principle, any model to be combined with any experimental procedure without requiring the procedure to be specifically designed for a given model.

*3. Iterate:* This final step in the ideal user workflow closes the loop between output of results and model construction. It can be used to define the differences in configuration between simulations belonging to the same simulation project, research project, educational tutorial or scientific publication. Further, this step can be automated in order to identify strengths, weaknesses, or deficits of a specific model. As such it becomes a powerful tool for pre-review of a model before its publication.

In this presentation we will demonstrate how G-3 interfaces to a variety of existing databases which are accordingly then linked to model development and simulation. In principle this capability should accelerate a shift from purely procedural to data-driven modeling with important consequences for computational modelling methodology, and especially new forms of model publication and scientific communication.
